# Acute and chronic impact of interleukin-33 stimulation on chemokines and growth factors in human cord blood-derived mast cells

**DOI:** 10.1371/journal.pone.0311981

**Published:** 2024-10-21

**Authors:** Sherin Bakhashab, Ghalya H. Banafea, Farid Ahmed, Reem Alsolami, Hans-Juergen Schulten, Kalamegam Gauthaman, Muhammad Imran Naseer, Peter Natesan Pushparaj

**Affiliations:** 1 Department of Biochemistry, King Abdulaziz University, Jeddah, Saudi Arabia; 2 Center of Excellence in Genomic Medicine Research, King Abdulaziz University, Jeddah, Saudi Arabia; 3 Department of Medical Laboratory Technology, Faculty of Applied Medical Sciences, King Abdulaziz University, Jeddah, Saudi Arabia; 4 Department of Pharmacology, Center for Transdisciplinary Research, Saveetha Dental College and Hospitals, Saveetha Institute of Medical and Technical Sciences, Saveetha University, Chennai, India; University of Tennessee Health Science Center, UNITED STATES OF AMERICA

## Abstract

**Background:**

Mast cells (MCs) are multifaceted immune cells that are capable of recognizing and responding to various stimuli by releasing an array of cytokines. We aimed to use human cord blood-derived mast cells (hCBMCs) as a model to evaluate different conditions under which chemokines and growth factors are expressed and secreted as mediators upon stimulation with the alarmin interleukin-33 (IL-33).

**Methods:**

hCBMCs were stimulated with 10 ng/mL or 20 ng/mL of recombinant human IL-33 (rhIL-33) for 6 h (acute) or 24 h (chronic). The mRNA expression of chemokines and growth factors was analyzed using microarrays, and the mediators released in the supernatant were evaluated using a multiplex assay.

**Results:**

The mRNA expression levels of C-C chemokine ligands (CCL) CCL1, CCL5, granulocyte macrophage colony-stimulating factor (GM-CSF), and macrophage inflammatory protein (MIP)-4/CCL18 were upregulated under all conditions. In contrast, C-X-C motif chemokine ligand (CXCL) CXCL8 and CCL24 levels increased only under acute (6 h) and prolonged (24 h) conditions, respectively. Moreover, high levels of CXCL8, MIP-1α, and MIP-1β were secreted during acute inflammation, whereas the release of GM-CSF and CXCL9 proteins increased under all four conditions.

**Conclusions:**

This study highlights the sentinel role of MCs in mounting a specific immune response against a pathogenic-like stimulus in a timely and dose-dependent manner and is relevant for improving inflammatory treatment options.

## Introduction

Mast cells (MCs) are immune cells that play a key role in connecting innate and acquired immune systems. In humans, MCs originate from the bone marrow cluster of differentiation (CD)34^+^ hematopoietic stem cells, circulate in the blood as CD117^+^ committed progenitors [1], and complete their differentiation into CD34^-^CD117^+^Fc Epsilon Receptor I (FcεRI)^low^ cells in the tissue [1, 2]. MCs are found in all tissues and their phenotypes are generally classified according to the combination of proteases contained within their granules. MCs found in connective tissue contain chymase and tryptase, whereas MCs in mucosal tissue only contain chymase. MCs play a key role in hypersensitivity reactions as they release numerous pro-inflammatory mediators (histamine, leukotrienes, and chemokines) in response to allergens through immunoglobulin E (IgE)-FcεRI crosslinking [3, 4]. In addition to their role in hypersensitivity, MCs recognize and respond to innate signals and cytokines.

Interleukin-33 (IL-33) belongs to the IL-1 family of pro-inflammatory cytokines. It is released from epithelial and endothelial cells following necrosis and activates MCs via interleukin 1 receptor-like 1 (IL1RL1)/ suppression of tumorigenicity 2 (ST2) surface receptors [5, 6]. IL-33 amplifies the inflammatory response of mast cells, leading to increased release of mediators. This effect was notably observed when mast cells activated by the severe acute respiratory syndrome coronavirus 2 (SARS-CoV-2) spike protein or complement peptides were co-stimulated with IL-33 [7, 8]. C-C chemokine ligands (CCL) CCL2 and CCL5 are released by MCs upon IL-33 stimulation, and thus contribute substantially to inflammation [9]. Both CCL2 and CCL5 promote the accumulation of macrophages and granulocytes in the airways of asthma patients, thereby increasing the severity of airway inflammation [10, 11].

However, a comprehensive understanding of the mediators released by MCs in response to IL-33 stimulation has yet to be achieved. This study leveraged high-throughput microarrays and multiplex enzyme-linked immunosorbent assay (ELISA) to evaluate chemokines and growth factors expressed and secreted by human cord blood-derived mast cells (hCBMCs) in response to acute and chronic stimulation with IL-33.

## Materials and methods

### Sample collection

Umbilical cord blood was collected from healthy donors after obtaining informed consent from December 02, 2020 to September 04, 2021. This study was approved by the Biomedical Ethics Unit, Faculty of Medicine, King Abdulaziz University (KAU, Approval Number 590–20). To yield sufficient cell numbers, each sample consisted of cord blood pooled from to 2–3 donors. CD34^+^ hematopoietic stem cells were isolated using Lymphoprep (1.077 g/ml, Axis Shield, Oslo, Norway), followed by CD34 microbead labelling and magnetic-activated cell sorting (Miltenyi Biotec Inc., Bergisch Gladbach, Germany).

### hCBMC culture and stimulation by IL-33

CD34^+^ hematopoietic stem cells were cultured in AIM-V medium (Thermo Fisher Scientific, Waltham, MA, USA) supplemented with recombinant human interleukin-6 (rhIL-6, 50 ng/mL; Thermo Fisher Scientific) and stem cell factor (rhSCF, 100 ng/mL; Miltenyi Biotec Inc.) for 8–10 weeks to support their differentiation. hCBMCs were characterized by MC phenotyping using the flow cytometric MC-specific markers BV421 mouse anti-human CD117, PerCP-Cy5.5 mouse anti-human CD23, APC mouse anti-human CD203c, FITC mouse anti-human CD45, and BV510 FCεR1α (BD Biosciences, Franklin Lakes, NJ, USA, S1 and S2 Figs); cell imaging using Giemsa and toluidine blue to visualize the granules; and gene set enrichment analysis to test possible dysregulation in molecular pathways, as previously reported [2]. hCBMCs were treated under four different conditions: stimulated with either 10 or 20 ng/mL recombinant human IL-33 (rhIL-33; Sino Biological, Beijing, China) and then incubated for 6 h or 24 h in a humidified cell culture chamber at 37°C with 5% CO_2_. The rhIL-33 concentrations used were in the range commonly used for assessing inflammatory effects. The two different incubation periods address immediate, acute, and prolonged inflammatory responses, respectively.

### Multiplex evaluation of chemokines and growth factors released by hCBMCs

The levels of chemokines and growth factors in hCBMC culture supernatants were measured using the Human Cytokine Magnetic 30-Plex Panel (Novex® Invitrogen, Thermo Fisher Scientific; Catalogue number: LHC6003M) according to the manufacturer’s guidelines and analyzed using the MAGPIX® instrument (Luminex Corporation, Austin, TX, USA). Each condition was set up in triplicate and each triplicate was measured four times.

### Total RNA isolation and microarray hybridization

Total RNA was isolated using the RNAeasy Mini Kit (Qiagen, Hilden, Germany), followed by on-column DNase digestion with the RNase-free DNase set (Qiagen) according to the manufacturer’s instructions. Two biological replicates for microarray experiments were assessed using Affymetrix Gene Chip Human Gene 1.0 ST arrays (Thermo Fisher Scientific), according to the manufacturer’s instructions, as previously described [2]. Subsequently, the arrays were scanned using an Affymetrix GeneChip® scanner 3000 7G, and the resulting raw CEL files were subjected to quality control and analyzed using Transcriptome Analysis Console (TAC) software (Thermo Fisher Scientific). The generated datasets were submitted to NCBI’s Gene Expression Omnibus (GEO) and are accessible under accession number GSE224089.

### Gene set enrichment analysis

The WEB-based Gene SeT AnaLysis Toolkit (WebGestalt) was used to perform the functional enrichment analysis [12]. Here, the gene set enrichment analysis (GSEA) method and Gene Ontology (GO) [13, 14] functional database were used to annotate the biological processes associated with a set of all 53 chemokines and growth factors detected in hCBMCs by Affymetrix Gene Chip Human Gene 1.0 ST arrays (Thermo Fisher Scientific). The parameters used for the analysis were as follows: the minimum number of IDs in each category was set to five, the maximum number of IDs was set to 2000 and the number of maximal permutations was set to 1000. The top five positively enriched categories were ranked based on p-value and enrichment score (ES).

### Statistical analysis

Transcriptome data were analyzed using TAC and are represented as Log2 fold change (FC) when samples or groups of samples were compared. Significance was calculated using TAC’s built-in empirical Bayes and was defined as FC ≥ 2 and p < 0.05.

The accuracy of the protein level data is represented as mean ± standard deviation (SD). The variance between the IL-33-induced groups and the control was calculated using two-way analysis of variance (ANOVA) followed by Tukey’s multiple comparisons test, and p < 0.05 was considered statistically significant. Statistical analyses were performed, and graphs were generated using GraphPad Prism 9.3.1 (GraphPad Software, San Diego, CA, USA).

## Results

### rhIL-33 induced the expression of chemokines in hCBMCs

The overall transcriptome of hCBMCs in response to rhIL-33 was evaluated and compared to that of untreated controls using Affymetrix microarray technology. Table 1 shows the differential expression of the chemokines at the mRNA level. rhIL-33 potently increased the mRNA expression of CCL1, CCL5, and CCL18 (FC > 2, p < 0.05) in both acute and prolonged conditions, represented by exposure to rhIL-33 for 6 and 24 h, respectively. Moreover, the mRNA expression of C-X-C motif chemokine ligand 8 (CXCL8/IL-8) was significantly elevated after 6 h of stimulation, while that of CCL24 was significantly elevated after 24 h.

**Table 1:** mRNA expression of chemokines in hCBMCs in response to acute and prolonged rhIL-33 stimulation.

	10 ng/mL for 6 h vs CTRL		10 ng/mL for 24 h vs CTRL		20 ng/mL for 6 h vs CTRL		20 ng/mL for 24 h vs CTRL	
Gene symbol	FC	P-value	FC	P-value	FC	P-value	FC	P-value
CCL1	28.08	8.69E-06***	25.88	1.06E-05***	34.88	5.29E-06***	33.72	5.70E-06***
CCL5	3.2	0.0252*	3.54	0.0172*	4.27	0.0087**	5.4	0.0038**
CCL11	−1.18	0.3768	−1.37	0.1068	−1.23	0.2748	−1.3	0.1704
CCL18; MIP-4	7.02	0.0146*	11.41	0.0046**	8.37	0.0095**	12.42	0.0037**
CCL24	2.67	0.1265	4.22	0.0362*	3.26	0.0738	4.22	0.0361*
CXCL8; IL-8	7.75	0.0151*	2.28	0.2562	9.57	0.0093**	2.7	0.1775
CXCL9	−1.02	0.8314	−1.04	0.7061	−1.05	0.6672	−1.04	0.6858

mRNA expression was analyzed using microarray and TAC software. Fold change (FC).

* p < 0.05

** p < 0.01

*** p < 0.001.

### hCBMCs released chemokines in response to rhIL-33

**Fig 1:**
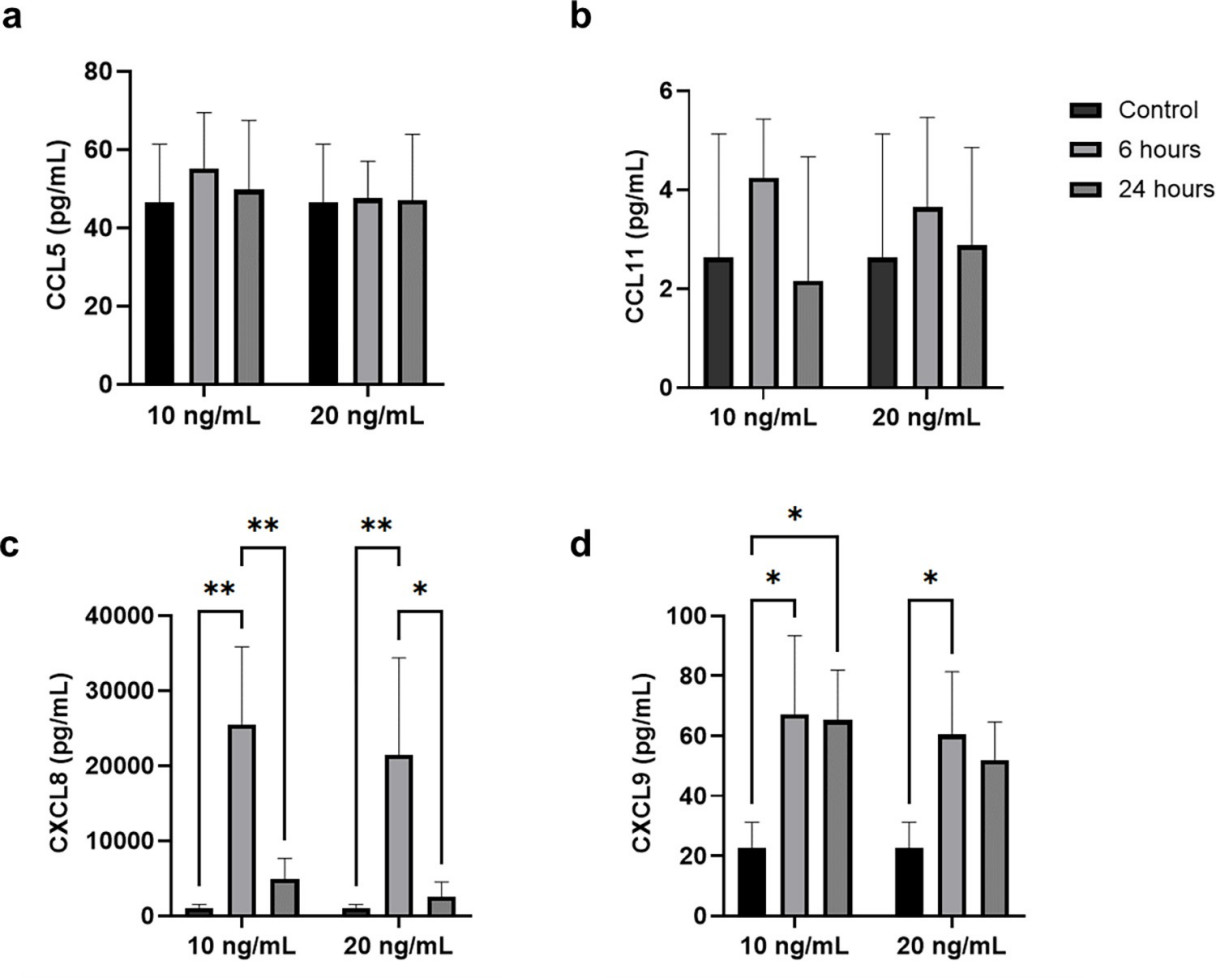
Concentrations of chemokines measured in the hCBMC supernatant via a multiplex xMAP assay after stimulation with 10 and 20 ng/mL of IL-33 for 6 and 24 h, in comparison to an untreated control. (a) CCL5. (b) CCL11/Eotaxin. (c) CXCL8/IL-8. (d) CXCL9. * p < 0.05, ** p < 0.01. All experiments were performed with four independent biological replicates.

Evaluation of the released chemokines in the supernatant of hCBMCs using a multiplex assay did not reveal a change in the release of CCL5 or CCL11, despite the increase in the former’s mRNA expression (Fig 1A and 1B); however, a potent increase in the release of CXCL8 was observed after stimulation with IL-33 for 6 h, which dropped significantly after 24 h of exposure (p < 0.05; Fig 1C). Moreover, CXCL9 was significantly elevated in the hCBMC supernatant after 6 h and 24 h of stimulation with IL-33 compared to the control (Fig 1D).

### Differential expression of growth factors and macrophage inflammatory chemokines by hCBMCs in response to rhIL-33

The mRNA expression of growth factors was analyzed, revealing an increase in the expression of colony-stimulating factor 2 (CSF2), encoding granulocyte-macrophage colony-stimulating factor (GM-CSF). However, no changes were detected in other growth factors, namely CSF3, epidermal growth factor (EGF), fibroblast growth factor 2 (FGF2), and vascular endothelial growth factor A (VEGFA). Furthermore, the mRNA expression of CCL18 encoding macrophage inflammatory protein-4 (MIP-4) was highly elevated upon both acute and prolonged exposure to rhIL-33, whereas no significant changes were detected in CCL3/MIP-1α, CCL4/MIP-1β, or monocyte chemoattractant protein-1 (CCL2/MCP-1; Table 2). Further, the mRNA expression data from microarray were summarized in S1 Table.

**Table 2:** mRNA expression of growth factors and macrophage inflammatory proteins in hCBMCs in response to acute and prolonged rhIL-33 stimulation.

	10 ng/mL for 6 h vs CTRL		10 ng/mL for 24 h vs CTRL		20 ng/mL for 6 h vs CTRL		20 ng/mL for 24 h vs CTRL	
Gene symbol	FC	P-value	FC	P-value	FC	P-value	FC	P-value
CSF2; GM-CSF	7.82	0.0031**	3.73	0.0298*	10.67	0.0013**	5.45	0.0091**
CSF3; G-CSF	−1.05	0.6813	−1.17	0.2397	−1.17	0.246	−1.27	0.082
EGF	−1.06	0.7435	1.03	0.8812	−1.12	0.5226	−1.11	0.5534
FGF2	−1	0.997	1.04	0.7391	−1.05	0.6308	1	0.997
VEGFA	1.66	0.0545	1.01	0.9518	2.13	0.0095	1.11	0.6606
CCL2; MCP-1	−1.01	0.9504	−1.51	0.1068	1.07	0.7788	−1.31	0.2703
CCL3; MIP-1a	1.77	0.2447	1.84	0.2198	1.76	0.2498	1.81	0.2308
CCL4; MIP-1b	2.72	0.3343	1.52	0.6794	3.48	0.236	1.9	0.53

mRNA expression was analysed using microarray and TAC software. Fold change (FC).

* p < 0.05

** p < 0.01

*** p < 0.001.

### hCBMCs release growth factors and macrophage inflammatory proteins in response to rhIL-33

**Fig 2:**
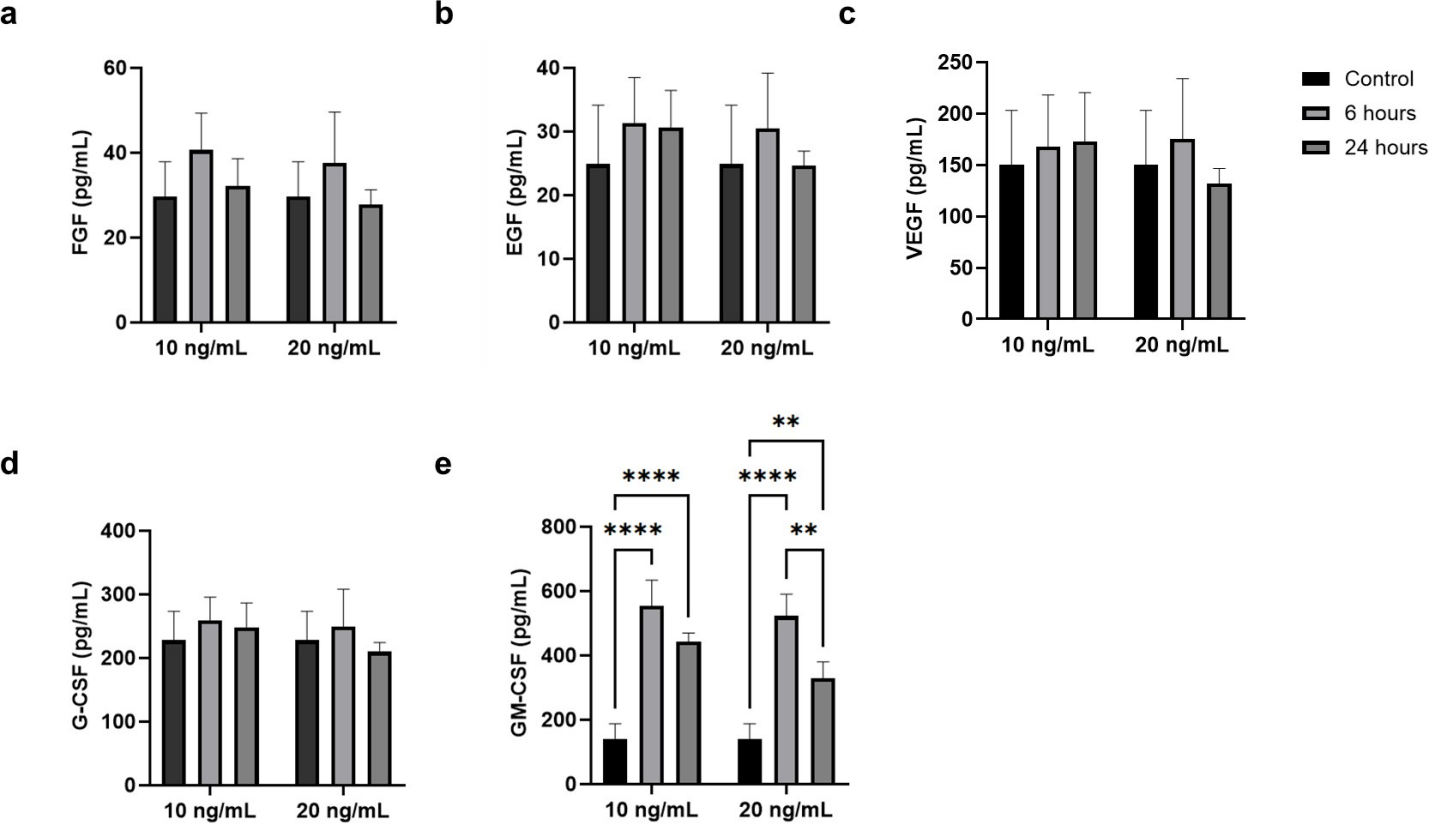
Concentrations of growth factors measured in hCBMC supernatant via a multiplex xMAP assay after stimulation with 10 and 20 ng/mL IL-33 for 6 and 24 h in comparison to an untreated control. (a) FGF-B. (b) EGF. (c) VEGF/VEGF-A ratio. (d) G-CSF/CSF3. (e) GM-CSF/CSF2. * p < 0.05, ** p < 0.01, *** p < 0.001, **** p < 0.0001. All experiments were performed in four independent biological replicates.

**Fig 3:**
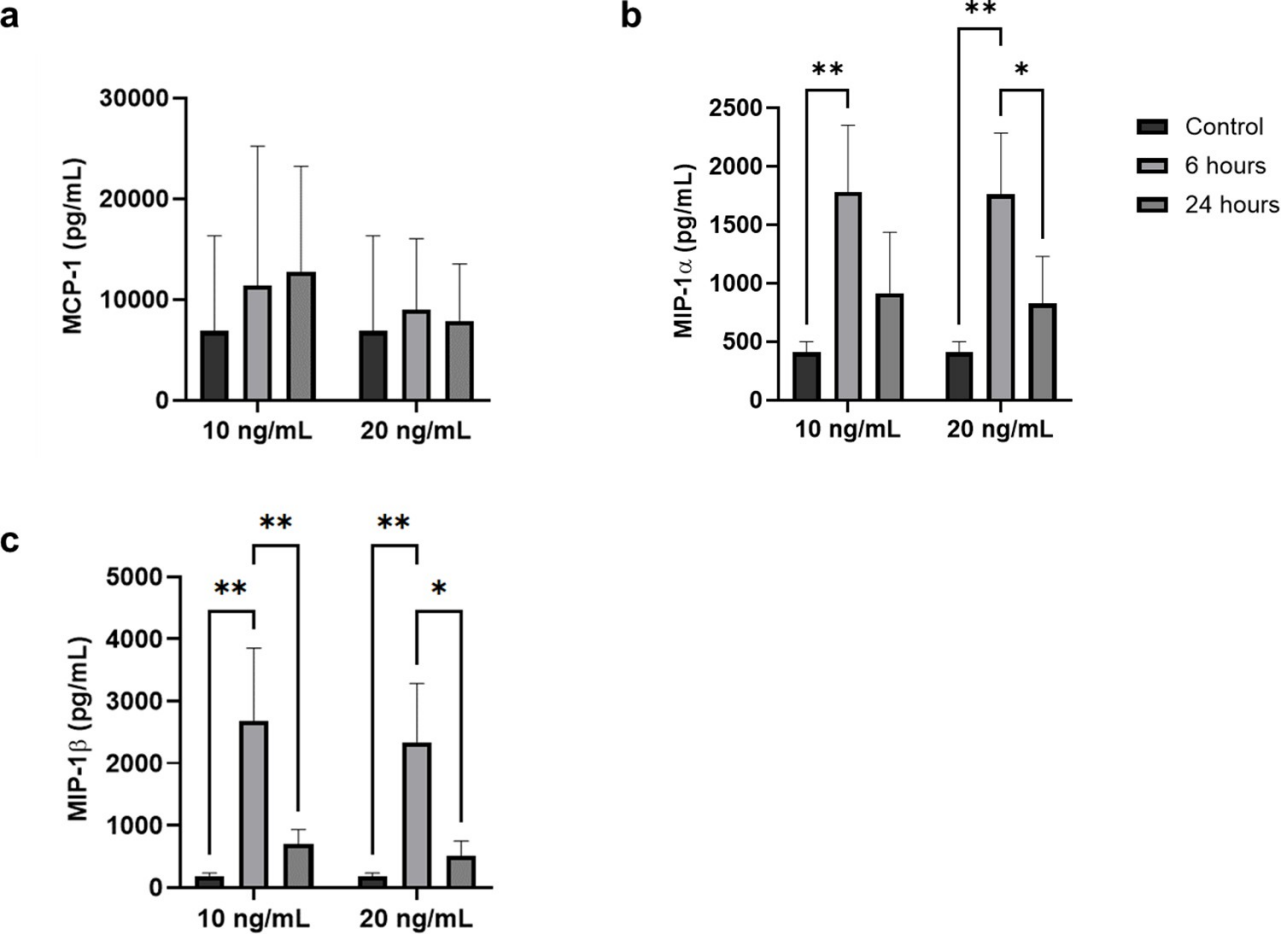
Concentrations of monocyte-targeting chemokines measured in hCBMCs’ supernatants via a multiplex xMAP assay after stimulation with 10 and 20 ng/mL IL-33 for 6 and 24 h, in comparison to an untreated control. (a) CCL2/MCP-1. (b) CCL3/MIP-1α. (c) CCL4/MIP-1β. * p < 0.05, ** p < 0.01. All experiments were performed in four independent biological replicates.

Evaluation of the supernatant of hCBMCs did not reveal a change in the levels of the analyzed growth factors FGF, EGF, VEGF, and G-CSF (Fig 2A–2D), except for GM-CSF, which increased in the supernatant after both 6 and 24 h of exposure to rhIL-33 (Fig 2E). Although the mRNA expression of CCL3/MIP-1α and CCL4/MIP-1β was not significantly altered in response to IL-33, a four-fold increase in CCL3/MIP-1α protein was detected in the supernatant of hCBMCs, accompanied by an over ten-fold increase in CCL4/MIP-1β protein levels after 6 h. Moreover, the increase in both MIPs decreased after 24 h of stimulation with IL-33 (Fig 3B and 3C), whereas no significant change was detected in the release of MCP-1 (Fig 3A).

### Biological processes enriched in hCBMCs in response to rhIL-33 stimulation

**Fig 4:**
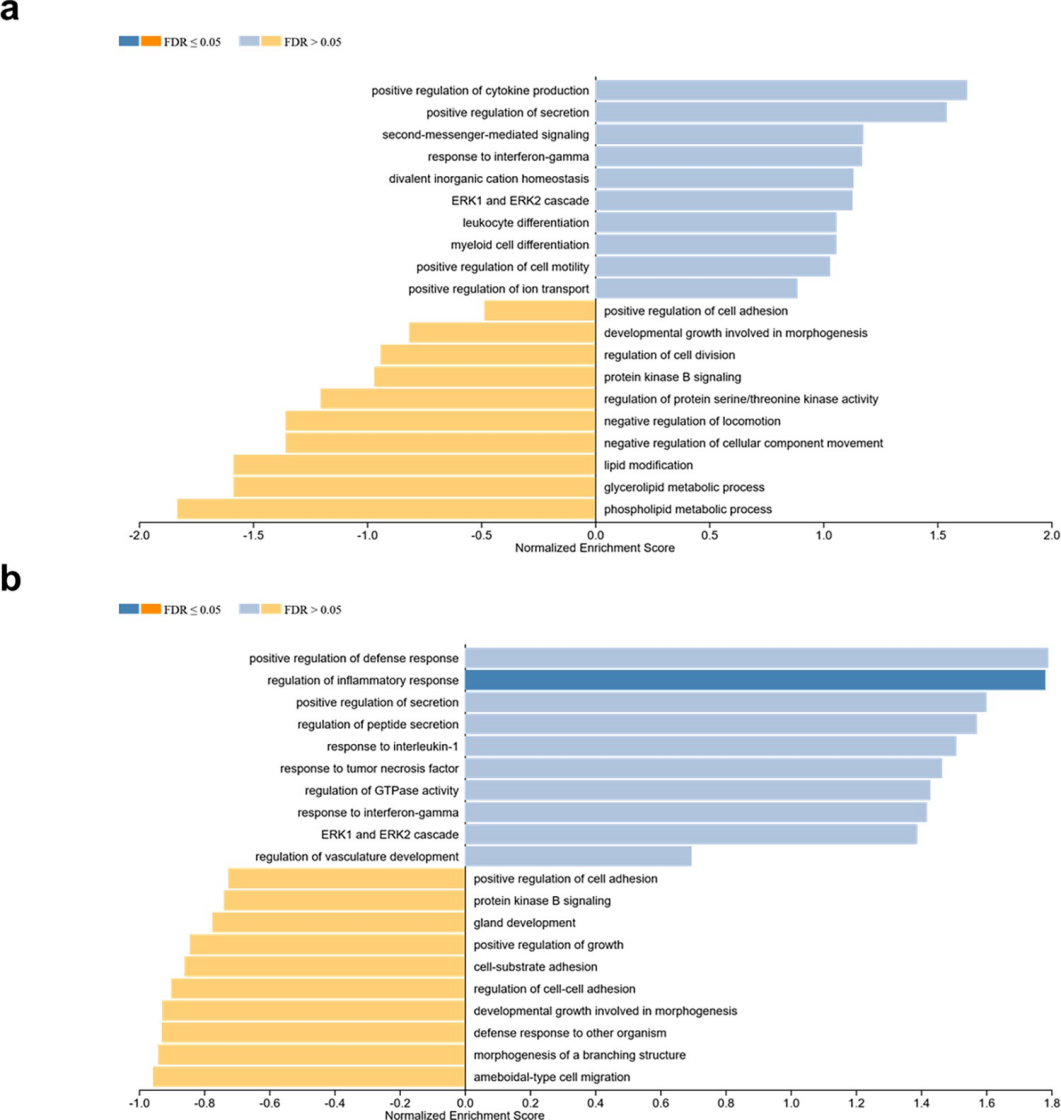
Biological processes enriched in hCBMCs stimulated with 10 ng rhIL-33 for (a) 6 h and (b) 24 h. The analysis was performed using the WebGestalt software. FDR: False discovery rate.

Enrichment analysis of chemokines and growth factors expressed by hCBMCs using WebGestalt revealed distinct biological processes that were enriched after acute and prolonged stimulation with rhIL-33. hCBMCs exposed to 10 ng of rhIL-33 for 6 h (Fig 4A) showed enrichment of the positive regulation of cytokine production GO:0001819 (ES = 0.901, p = 0.012), whereas hCBMCs exposed for 24 h (Fig 4B) displayed enrichment of the positive regulation of the defense response GO:0031349 (ES = 0.892, p = 0.001), regulation of the inflammatory response GO:0050727 (ES = 0.870, p = 0.003), response to IL-1 GO:0070555 (ES = 0.669, p = 0.03), and response to tumor necrosis factor GO:0034612 (ES = 0.646, p = 0.04).

**Fig 5:**
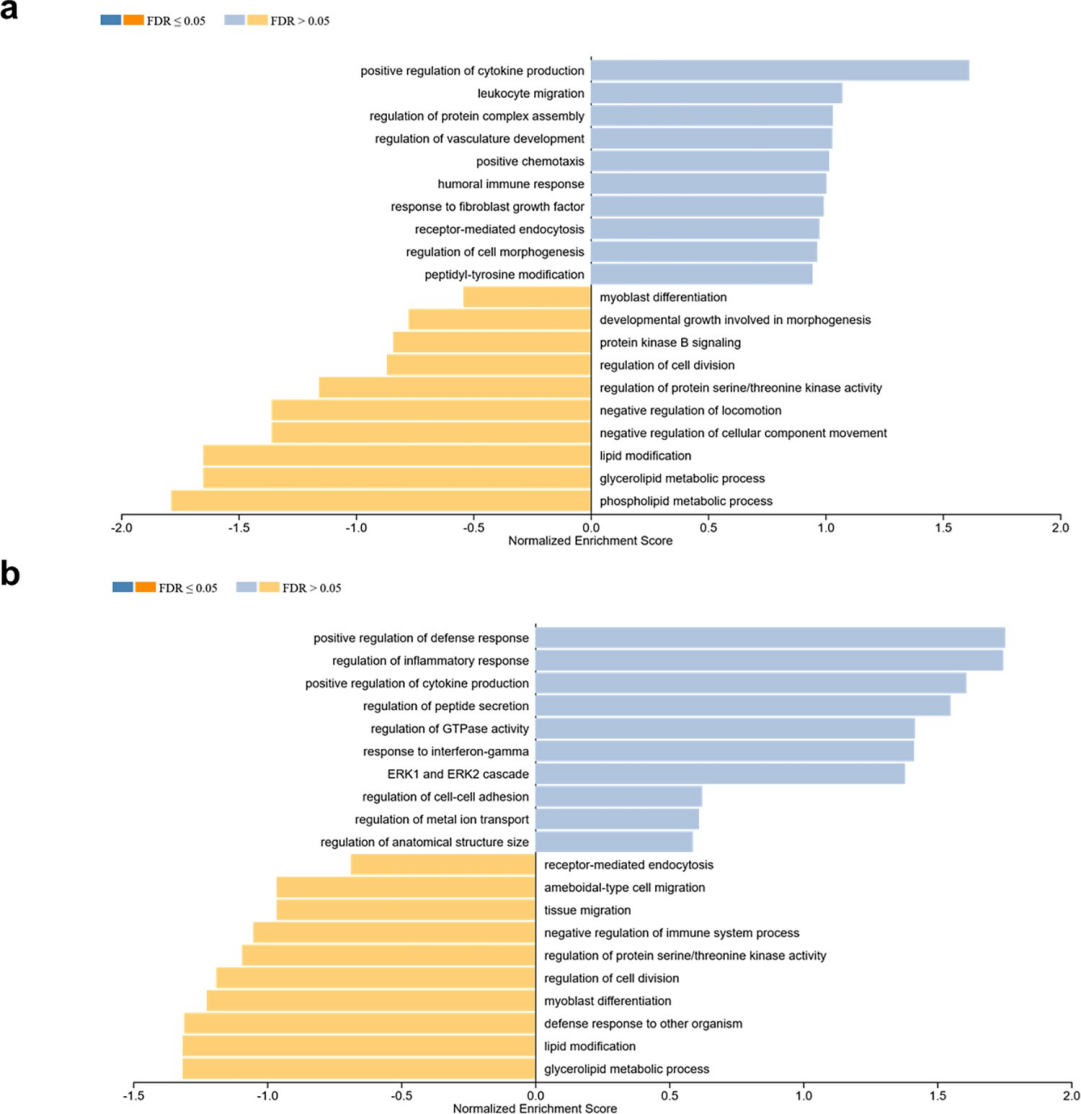
Biological processes enriched in hCBMCs stimulated with 20 ng rhIL-33 for (a) 6 h and (b) 24 h. Analysis was performed using WebGestalt. FDR: False discovery rate.

A similar pattern was observed when hCBMCs were exposed to 20 ng rhIL-33. hCBMCs exposed for 6 h (Fig 5A) showed enrichment of positive regulation of cytokine production (ES = 0.915, p = 0.01), whereas cells exposed for 24 h (Fig 5B) showed enrichment of positive regulation of the defense response (ES = 0.904, p = 0.003), regulation of the inflammatory response (ES = 0.886, p = 0.005), and positive regulation of cytokine production (ES = 0.88, p = 0.04).

## Discussion

This study sheds light on the interplay between MCs and IL-33 expression. These findings contribute to our understanding of the roles of MCs and IL-33 in chemotaxis and immune cell polarization during inflammation.

The release of chemokines by MCs has been previously evaluated by Emi-Sugie et al. [15] in the mucosal MC phenotype derived from peripheral blood. Our previous study analyzed cytokines released by the connective tissue MC phenotype derived from human cord blood CD34^+^ progenitors [2, 16]. To obtain a complementary picture, this study evaluated the release of chemokines and growth factors.

MCs have previously been linked to eosinophilic asthma [17], and CCL24 (Eotaxin-2) is known to augment inflammation by recruiting eosinophils to the airways [18]. CCL24 was significantly increased after prolonged stimulation with rhIL-33.

Furthermore, MCs are essential for defending the body and clearing viral and parasitic infections [19, 20]. rhIL-33 elicited a strong increase in the mRNA expression of CCL1, a chemokine essential for mounting an effective immune response against helminths by promoting the survival and proliferation of group 2 innate lymphoid cells [21]. MCs also release CXCL9 upon acute stimulation with rhIL-33, which is crucial for efficient pathogen clearance because it recruits CD4^+^ and CD8^+^ T cells [22]. Moreover, CXCL8 (IL-8) levels were dramatically increased. It is also notable that this potent neutrophil chemoattractant and activator showed a significant decrease after 24 h, which aligns with the pivotal function of neutrophils in rapidly mounting an immune response against viruses and bacteria [23–25].

In addition to chemokines, MCs can produce an array of growth factors, the expression and release of which was assessed in this study in IL-33-activated MCs. GM-CSF is a growth factor that promotes the migration and responsiveness of eosinophils, neutrophils and monocytes [26–28]. The upregulation of GM-CSF is strongly associated with acute conditions. However, the upregulation of CCL18/MIP-4 was more strongly associated with prolonged exposure. CCL18/MIP-4 recruits type-2 T helper cells (Th2) and basophils and induces mediator release [29].

Moreover, IL-33-activated MCs are associated with macrophage infiltration in gastric tumors [30]. Stimulation of hCBMCs with rhIL-33 induced drastic release of CCL3/MIP-1α and CCL4/MIP-1β within 6 h. CCL3/MIP-1α promotes monocyte chemotaxis and polarization into the proinflammatory M1 subtype [31, 32]. In contrast, CCL4/MIP-1β promotes eosinophil chemotaxis [33]. In patients with asthma, monocytes are recruited into the airways by the actions of CCL5 [34], which was increased in our study at the mRNA level; however, no notable change was observed at the released protein level, suggesting distinct modulation of its mRNA expression and protein release.

Notably, we observed a mismatch between mRNA and secreted protein levels of a number of mediators, namely CCL1, CCL5, CCL24, CXCL8, CXCL9, CCL3/MIP-1α, and CCL4/MIP-1β. Secreted proteins have been previously reported to exhibit imperfect correlation with mRNA levels, which can be explained by post-transcriptional regulation, individual sequences, and gene class characteristics [35, 36].

WebGestalt analysis revealed a distinct response of hCBMCs to IL-33 under acute and prolonged stimulation. Acute stimulation, represented by exposure for 6 h, enriched the positive regulation of cytokine production. This early response is consistent with IL-33’s role as a proinflammatory cytokine capable of stimulating the immune response [37]. However, after prolonged stimulation for 24 h, the enrichment shifted towards processes associated with the defense response, a crucial function of MCs [19, 20], followed by regulation of the inflammatory response, including modulation of inflammation, which was also portrayed by the decreased production of CXCL8, GM-CSF, CCL3/MIP-1α, and CCL4/ MIP-1β upon prolonged exposure to rhIL-33. These findings highlight the multifaceted nature of the response of hCBMCs to rhIL-33 and the importance of considering the duration of IL-33 exposure when studying the immune response in MCs.

## Conclusions

In conclusion, this study highlights various mediators released by hCBMCs in response to rhIL-33 stimulation. Chemokines and growth factors released by IL-33-activated MCs are essential for mounting an effective immune response against pathogens; however, they have also been implicated in exacerbating inflammation. Therefore, these findings not only advance our understanding of MCs but also pave the way for exploring potential therapeutic targets for inflammation.

## Supporting information

S1 Fig The purity of the CD34^+^ hematopoietic stem cells was > 90% before initiating the differentiation protocol. (PDF)

S2 Fig Characterization of human cord blood derived mast cells (hCBMCs) by flow cytometry. FACS analysis of hCBMCs after 8 weeks of differentiation using MC cell surface markers FcεRIα, CD117, CD45, CD23, and CD34.
(PDF)

S1 Table mRNA expression of mast cell chemokines and growth factors in hCBMCs in response to acute and prolonged rhIL-33 stimulation. (PDF)

## References

[1] Dahlin JS, Malinovschi A, Öhrvik H, Sandelin M, Janson C, Alving K, et al. Lin- CD34hi CD117int/hi FcεRI+ cells in human blood constitute a rare population of mast cell progenitors. Blood. 2016;127(4):383–91. Epub 20151201. doi: 10.1182/blood-2015-06-650648 ; PubMed Central PMCID: PMC4731844. 26626992 PMC4731844

[2] Bakhashab S, Banafea GH, Ahmed F, Alsehli H, AlShaibi HF, Bagatian N, et al. Characterization of human umbilical cord blood-derived mast cells using high-throughput expression profiling and next-generation knowledge discovery platforms. Experimental and Molecular Pathology. 2023;132–133:104867. doi: 10.1016/j.yexmp.2023.104867 37634863

[3] Cruse G, Kaur D, Yang W, Duffy SM, Brightling CE, Bradding P. Activation of human lung mast cells by monomeric immunoglobulin E. European Respiratory Journal. 2005;25(5):858–63. doi: 10.1183/09031936.05.00091704 15863643

[4] Jayapal M, Tay HK, Reghunathan R, Zhi L, Chow KK, Rauff M, et al. Genome-wide gene expression profiling of human mast cells stimulated by IgE or FcεRI-aggregation reveals a complex network of genes involved in inflammatory responses. BMC Genomics. 2006;7(1):210. 10.1186/1471-2164-7-210. 16911805 PMC1564015

[5] Schmitz J, Owyang A, Oldham E, Song Y, Murphy E, McClanahan TK, et al. IL-33, an interleukin-1-like cytokine that signals via the IL-1 receptor-related protein ST2 and induces T helper type 2-associated cytokines. Immunity. 2005;23(5):479–90. doi: 10.1016/j.immuni.2005.09.015 . 16286016

[6] Travers J, Rochman M, Miracle CE, Habel JE, Brusilovsky M, Caldwell JM, et al. Chromatin regulates IL-33 release and extracellular cytokine activity. Nature Communications. 2018;9(1):3244. doi: 10.1038/s41467-018-05485-x 30108214 PMC6092330

[7] Tsilioni I, Theoharides TC. Recombinant SARS-CoV-2 Spike Protein Stimulates Secretion of Chymase, Tryptase, and IL-1β from Human Mast Cells, Augmented by IL-33 International Journal of Molecular Sciences. 2023;24(11):9487. doi: 10.3390/ijms24119487 37298438 PMC10253625

[8] West PW, Bahri R, Garcia-Rodriguez KM, Sweetland G, Wileman G, Shah R, et al. Interleukin-33 Amplifies Human Mast Cell Activities Induced by Complement Anaphylatoxins. Frontiers in Immunology. 2021;11. doi: 10.3389/fimmu.2020.615236 33597949 PMC7882629

[9] Bawazeer MA, Theoharides TC. IL-33 stimulates human mast cell release of CCL5 and CCL2 via MAPK and NF-κB, inhibited by methoxyluteolin. European Journal of Pharmacology. 2019;865:172760. 10.1016/j.ejphar.2019.172760. 31669588

[10] Gauthier M, Kale S, Oriss T, Gorry M, Ramonell R, Scholl K, et al. CCL5 is a Potential Bridge Between Type 1 and Type 2 Inflammation in Asthma. Journal of Allergy and Clinical Immunology. 2023;151(2):AB224. doi: 10.1016/j.jaci.2023.02.028 36893862 PMC10330021

[11] Lee YG, Jeong JJ, Nyenhuis S, Berdyshev E, Chung S, Ranjan R, et al. Recruited alveolar macrophages, in response to airway epithelial-derived monocyte chemoattractant protein 1/CCl2, regulate airway inflammation and remodeling in allergic asthma. Am J Respir Cell Mol Biol. 2015;52(6):772–84. doi: 10.1165/rcmb.2014-0255OC ; PubMed Central PMCID: PMC4491131. 25360868 PMC4491131

[12] Liao Y, Wang J, Jaehnig EJ, Shi Z, Zhang B. WebGestalt 2019: gene set analysis toolkit with revamped UIs and APIs. Nucleic Acids Research. 2019;47(W1):W199–W205. doi: 10.1093/nar/gkz401 31114916 PMC6602449

[13] Ashburner M, Ball CA, Blake JA, Botstein D, Butler H, Cherry JM, et al. Gene Ontology: tool for the unification of biology. Nature Genetics. 2000;25(1):25–9. doi: 10.1038/75556 10802651 PMC3037419

[14] Consortium TGO, Aleksander SA, Balhoff J, Carbon S, Cherry JM, Drabkin HJ, et al. The Gene Ontology knowledgebase in 2023. Genetics. 2023;224(1). doi: 10.1093/genetics/iyad031 36866529 PMC10158837

[15] Emi-Sugie M, Saito H, Matsumoto K. Cultured human mast cells release various chemokines after stimulation with IL-33. Allergology International. 2021;70(3):386–8. doi: 10.1016/j.alit.2020.12.002 33583698

[16] Bakhashab S, Banafea GH, Ahmed F, Alsolami R, Schulten H-J, Gauthaman K, et al. Acute and prolonged effects of interleukin-33 on cytokines in human cord blood-derived mast cells. Immunology Letters. 2024;269:106908. doi: 10.1016/j.imlet.2024.106908 39151731

[17] Wang RM, Mesfin JM, Karkanitsa M, Ungerleider JL, Zelus E, Zhang Y, et al. Immunomodulatory contribution of mast cells to the regenerative biomaterial microenvironment. npj Regenerative Medicine. 2023;8(1):53. doi: 10.1038/s41536-023-00324-0 37730736 PMC10511634

[18] Dai C, Yao X, Gordon EM, Barochia A, Cuento RA, Kaler M, et al. A CCL24-dependent pathway augments eosinophilic airway inflammation in house dust mite-challenged Cd163(-/-) mice. Mucosal Immunol. 2016;9(3):702–17. Epub 20150916. doi: 10.1038/mi.2015.94 ; PubMed Central PMCID: PMC4794428. 26376364 PMC4794428

[19] Hendriksen E, van Bergeijk D, Oosting RS, Redegeld FA. Mast cells in neuroinflammation and brain disorders. Neuroscience & Biobehavioral Reviews. 2017;79:119–33. 10.1016/j.neubiorev.2017.05.001. 28499503

[20] Shimokawa C, Kanaya T, Hachisuka M, Ishiwata K, Hisaeda H, Kurashima Y, et al. Mast Cells Are Crucial for Induction of Group 2 Innate Lymphoid Cells and Clearance of Helminth Infections. Immunity. 2017;46(5):863–74.e4. doi: 10.1016/j.immuni.2017.04.017 28514691

[21] Knipfer L, Schulz-Kuhnt A, Kindermann M, Greif V, Symowski C, Voehringer D, et al. A CCL1/CCR8-dependent feed-forward mechanism drives ILC2 functions in type 2-mediated inflammation. J Exp Med. 2019;216(12):2763–77. Epub 20190919. doi: 10.1084/jem.20182111 ; PubMed Central PMCID: PMC6888976. 31537642 PMC6888976

[22] Ochiai E, Sa Q, Brogli M, Kudo T, Wang X, Dubey JP, et al. CXCL9 is important for recruiting immune T cells into the brain and inducing an accumulation of the T cells to the areas of tachyzoite proliferation to prevent reactivation of chronic cerebral infection with Toxoplasma gondii. Am J Pathol. 2015;185(2):314–24. Epub 20141126. doi: 10.1016/j.ajpath.2014.10.003 ; PubMed Central PMCID: PMC4305179. 25432064 PMC4305179

[23] Bernhard S, Hug S, Stratmann AEP, Erber M, Vidoni L, Knapp CL, et al. Interleukin 8 Elicits Rapid Physiological Changes in Neutrophils That Are Altered by Inflammatory Conditions. Journal of Innate Immunity. 2021;13(4):225–41. doi: 10.1159/000514885 33857948 PMC8460987

[24] Ma Y, Zhang Y, Zhu L. Role of neutrophils in acute viral infection. Immun Inflamm Dis. 2021;9(4):1186–96. Epub 20210902. doi: 10.1002/iid3.500 ; PubMed Central PMCID: PMC8589350. 34472718 PMC8589350

[25] Witter AR, Okunnu BM, Berg RE. The Essential Role of Neutrophils during Infection with the Intracellular Bacterial Pathogen Listeria monocytogenes. J Immunol. 2016;197(5):1557–65. doi: 10.4049/jimmunol.1600599 ; PubMed Central PMCID: PMC4995063. 27543669 PMC4995063

[26] Griseri T, Arnold Isabelle C, Pearson C, Krausgruber T, Schiering C, Franchini F, et al. Granulocyte Macrophage Colony-Stimulating Factor-Activated Eosinophils Promote Interleukin-23 Driven Chronic Colitis. Immunity. 2015;43(1):187–99. doi: 10.1016/j.immuni.2015.07.008 26200014 PMC4518500

[27] Pinder EM, Rostron AJ, Hellyer TP, Ruchaud-Sparagano M-H, Scott J, Macfarlane JG, et al. Randomised controlled trial of GM-CSF in critically ill patients with impaired neutrophil phagocytosis. Thorax. 2018;73(10):918–25. doi: 10.1136/thoraxjnl-2017-211323 30064991 PMC6166597

[28] Vogel DYS, Kooij G, Heijnen PDAM, Breur M, Peferoen van der Valk P et al. GM-CSF promotes migration of human monocytes across the blood brain barrier. European Journal of Immunology. 2015;45(6):1808–19. 10.1002/eji.201444960. 25756873

[29] Nadaï Pd, Charbonnier A-S, Chenivesse Cc, Sänächal Sp, Fournier Cm, Gilet J, et al. Involvement of CCL18 in Allergic Asthma1. The Journal of Immunology. 2006;176(10):6286–93. 10.4049/jimmunol.176.10.6286. 16670340

[30] Eissmann MF, Dijkstra C, Jarnicki A, Phesse T, Brunnberg J, Poh AR, et al. IL-33-mediated mast cell activation promotes gastric cancer through macrophage mobilization. Nature Communications. 2019;10(1):2735. doi: 10.1038/s41467-019-10676-1 31227713 PMC6588585

[31] Yuan X, Liu W, Li Y, Chen K, Li H, Tang H, et al. CCL3 aggravates intestinal damage in NEC by promoting macrophage chemotaxis and M1 macrophage polarization. Pediatric Research. 2023;94(1):119–28. 10.1038/s41390-022-02409-w. 36550354

[32] Zhao X, Gu M, Xu X, Wen X, Yang G, Li L, et al. CCL3/CCR1 mediates CD14+CD16− circulating monocyte recruitment in knee osteoarthritis progression. Osteoarthritis and Cartilage. 2020;28(5):613–25. doi: 10.1016/j.joca.2020.01.009 32006659

[33] Kobayashi Y, Konno Y, Kanda A, Yamada Y, Yasuba H, Sakata Y, et al. Critical role of CCL4 in eosinophil recruitment into the airway. Clinical & Experimental Allergy. 2019;49(6):853–60. doi: 10.1111/cea.13382 30854716

[34] Allard B, Levardon H, Esteves P, Celle A, Maurat E, Thumerel M, et al. Asthmatic Bronchial Smooth Muscle Increases CCL5-Dependent Monocyte Migration in Response to Rhinovirus-Infected Epithelium. Front Immunol. 2019;10:2998. Epub 20200106. doi: 10.3389/fimmu.2019.02998 ; PubMed Central PMCID: PMC6956660. 31969885 PMC6956660

[35] Csárdi G, Franks A, Choi DS, Airoldi EM, Drummond DA. Accounting for Experimental Noise Reveals That mRNA Levels, Amplified by Post-Transcriptional Processes, Largely Determine Steady-State Protein Levels in Yeast. PLOS Genetics. 2015;11(5):e1005206. 10.1371/journal.pgen.1005206. 25950722 PMC4423881

[36] Nicolet BP, Wolkers MC. The relationship of mRNA with protein expression in CD8+ T cells associates with gene class and gene characteristics. PLoS One. 2022;17(10):e0276294. Epub 20221019. doi: 10.1371/journal.pone.0276294 ; PubMed Central PMCID: PMC9581405. 36260607 PMC9581405

[37] Chan BCL, Lam CWK, Tam L-S, Wong CK. IL33: Roles in Allergic Inflammation and Therapeutic Perspectives. Frontiers in Immunology. 2019;10. doi: 10.3389/fimmu.2019.00010 30886621 PMC6409346

