# Supplementary material for: Acute and chronic impact of interleukin-33 stimulation on chemokines and growth factors in human cord blood-derived mast cells
Source: PLoS One. 2024 Oct 21;19(10):e0311981. doi: 10.1371/journal.pone.0311981 (PMC11493263; doi:10.1371/journal.pone.0311981)
Supplement: S1 Fig — (PDF) [file pone.0311981.s001.pdf]

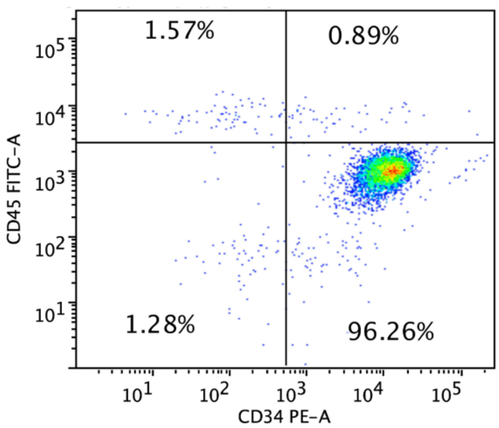

S1 Fig. The purity of the CD34$^+$ hematopoietic stem cells was > 90% before initiating the differentiation protocol.
