# Supplementary material for: Acute and chronic impact of interleukin-33 stimulation on chemokines and growth factors in human cord blood-derived mast cells
Source: PLoS One. 2024 Oct 21;19(10):e0311981. doi: 10.1371/journal.pone.0311981 (PMC11493263; doi:10.1371/journal.pone.0311981)
Supplement: S2 Fig — FACS analysis of hCBMCs after 8 weeks of differentiation using MC cell surface markers FcεRIα, CD117, CD45, CD23, and CD34. (PDF) [file pone.0311981.s002.pdf]

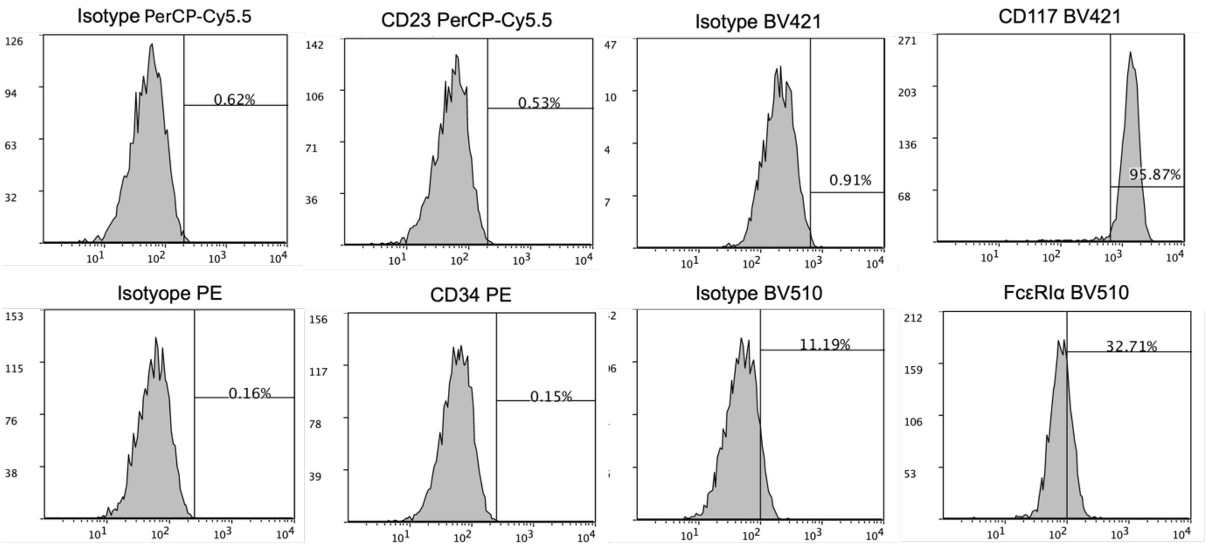

S2 Fig. Characterization of human cord blood derived mast cells (hCBMCs) by Flow Cytometry. FACS analysis of hCBMCs after 8 weeks of differentiation using MC cell surface markers FcεRIα, CD117, CD45, CD23, and CD34.
