# Supplementary material for: Acute and chronic impact of interleukin-33 stimulation on chemokines and growth factors in human cord blood-derived mast cells
Source: PLoS One. 2024 Oct 21;19(10):e0311981. doi: 10.1371/journal.pone.0311981 (PMC11493263; doi:10.1371/journal.pone.0311981)
Supplement: S1 Table — (PDF) [file pone.0311981.s003.pdf]

S1 Table. mRNA expression of mast cell chemokines and growth factors in hCBMCs in response to acute and prolonged rhIL-33 stimulation.

	10 ng/mL for 6 h vs CTRL		10 ng/mL for 24 h vs CTRL		20 ng/mL for 6 h vs CTRL		20 ng/mL for 24 h vs CTRL	
Gene symbol	FC	P-value	FC	P-value	FC	P-value	FC	P-value
CCL1	28.08	8.69E-06	25.88	1.06E-05	34.88	5.29E-06	33.72	5.70E-06
CCL2	-1.01	0.9504	-1.51	0.1068	1.07	0.7788	-1.31	0.2703
CCL3	1.77	0.2447	1.84	0.2198	1.76	0.2498	1.81	0.2308
CCL3L3	1.39	0.5668	1.29	0.6586	1.32	0.621	1.46	0.5086
CCL4	2.72	0.3343	1.52	0.6794	3.48	0.236	1.9	0.53
CCL4L2	4.33	0.1551	2.22	0.4187	5.18	0.1158	3.01	0.273
CCL5	3.2	0.0252	3.54	0.0172	4.27	0.0087	5.4	0.0038
CCL7	1.23	0.3027	1.4	0.1142	1.45	0.0875	1.12	0.5752
CCL8	1.41	0.0615	1.05	0.7614	1.33	0.1133	1.07	0.6656
CCL11	-1.18	0.3768	-1.37	0.1068	-1.23	0.2748	-1.3	0.1704
CCL13	1.75	0.3291	1.66	0.3745	2.25	0.1709	1.82	0.3013
CCL14	-1.16	0.1838	-1.09	0.414	-1.2	0.1088	-1.17	0.1633
CCL16	-1.01	0.9332	-1.09	0.4672	-1.05	0.6762	-1.06	0.6536
CCL17	1.09	0.5375	-1.02	0.8718	1.03	0.8062	1	0.9955
CCL18	7.02	0.0146	11.41	0.0046	8.37	0.0095	12.42	0.0037
CCL19	-1.09	0.5363	-1.17	0.2541	-1.22	0.1606	-1.3	0.0735
CCL20	-1.02	0.9312	-1.24	0.4093	1.09	0.7484	-1.45	0.1711
CCL21	1.05	0.6431	-1.03	0.7843	-1.01	0.9443	-1.07	0.5462
CCL22	1.46	0.5528	1.69	0.4135	1.67	0.4204	2.08	0.262
CCL23	-1.03	0.7507	-1.08	0.3862	-1	0.9572	1.08	0.3561
CCL24	2.67	0.1265	4.22	0.0362	3.26	0.0738	4.22	0.0361
CCL25	-1.05	0.6626	-1.22	0.1094	-1.08	0.5194	-1.14	0.2479
CCL26	1.01	0.9435	1.05	0.7233	-1.02	0.9008	1.05	0.7118

CCL27	-1.09	0.4456	-1.15	0.2362	-1.23	0.0951	-1.25	0.0767
CCL28	-1.64	0.1857	-1.46	0.3012	-1.5	0.2708	-1.52	0.2543
CSF1	1.16	0.5685	1.18	0.5276	1.28	0.3463	1.21	0.4602
CSF2	7.82	0.0031	3.73	0.0298	10.67	0.0013	5.45	0.0091
CSF3	-1.05	0.6813	-1.17	0.2397	-1.17	0.246	-1.27	0.082
FGF1	-1.21	0.0715	-1.25	0.0405	-1.25	0.0385	-1.2	0.0783
FGF2	-1	0.997	1.04	0.7391	-1.05	0.6308	1	0.997
FGF3	-1.08	0.4758	-1.13	0.2559	-1.18	0.1244	-1.16	0.1636
FGF4	-1.21	0.0429	-1.17	0.0956	-1.3	0.0113	-1.18	0.0795
FGF4	1.13	0.2	-1.07	0.4593	1.09	0.3434	-1.01	0.9278
FGF5	1.02	0.8396	1.03	0.7897	-1.03	0.7776	-1.11	0.3434
FGF6	-1.25	0.1114	-1.26	0.1011	-1.22	0.1427	-1.36	0.0369
FGF7	1.11	0.4434	1.19	0.2159	1.11	0.4541	1.13	0.3648
CXCL1	1.32	0.1988	1.15	0.5021	1.41	0.1167	1.03	0.8702
CXCL2	3.9	0.1277	1.22	0.8128	4.07	0.1176	1.32	0.7388
CXCL3	1.05	0.6795	1.01	0.9147	1.21	0.1514	-1.1	0.4779
CXCL5	1.3	0.0632	1.79	0.0014	1.46	0.0146	1.49	0.0118
CXCL6	-1.09	0.343	-1.05	0.5779	-1.1	0.3192	-1.06	0.5341
CXCL8	7.75	0.0151	2.28	0.2562	9.57	0.0093	2.7	0.1775
CXCL9	-1.02	0.8314	-1.04	0.7061	-1.05	0.6672	-1.04	0.6858
CXCL10	2.7	0.168	-1.07	0.9169	2.31	0.238	-1.1	0.8876
CXCL11	1.12	0.5605	-1.2	0.3692	1.06	0.7756	-1.22	0.3327
CXCL12	1.13	0.5286	-1.04	0.8583	-1.13	0.5236	-1.09	0.6444
CXCL13	-1.06	0.6047	1.15	0.2573	1.14	0.2852	-1.07	0.5712
CXCL14	-1.05	0.6712	-1.14	0.2509	-1.24	0.0795	-1.19	0.138
CXCL16	-1.67	0.0108	-1.7	0.0091	-1.58	0.0189	-1.65	0.0125

CXCL17	-1.08	0.4275	-1.2	0.0683	-1.25	0.0316	-1.16	0.135
VEGFA	1.66	0.0545	1.01	0.9518	2.13	0.0095	1.11	0.6606
VEGFB	-1.13	0.5226	-1.11	0.5744	-1.02	0.9064	-1.03	0.8778
VEGFC	1.32	0.2544	-1.16	0.5231	1.32	0.2515	-1.04	0.8817
EGF	-1.06	0.7435	1.03	0.8812	-1.12	0.5226	-1.11	0.5534

mRNA expression was analyzed using microarray and TAC software. Fold change (FC). * $p < 0.05$, ** $p < 0.01$, *** $p < 0.001$.
